# The moderating effect of information channel on the relationship between type of information search and knowledge of genetically modified organisms

**DOI:** 10.1080/21645698.2021.2015272

**Published:** 2022-01-30

**Authors:** Su-Jung Nam, Bumkyu Lee

**Affiliations:** a Department of Home Economics Education, College of Education, Jeonju University, Jeonju, Republic of Korea; b Department of Environmental Science & Biotechnology, College of Medical Science, Jeonju University, Jeonju, Republic of Korea

**Keywords:** GMO knowledge, active information seeker, passive information seeker, information channel

## Abstract

This study explores how the type of information search and information channel can influence the objective knowledge of consumers on genetically modified organisms. We divided the types of information search on genetically modified organisms into active and passive seekers, and then examined how their knowledge differed depending on preferred the information channel (i.e., government, portals, non-government organization (NGO) sites). An online survey was conducted with Korean men and women aged 19 or older. The main and interaction effects of the type of information search, and government, portal, and NGO sites were statistically significant. The results showed that active information seekers who prefer government, portal, and NGO sites have lower scores of knowledge on genetically modified organisms than that of passive information seekers, given the confusion of competing and sometimes inaccurate information sources.

## Introduction

Genetically modified organisms (GMOs) are organisms developed for special purposes, using modern biotechnologies to overcome the limitations of traditional breeding techniques.^1^ Genetically modified (GM) crops have steadily increased in cultivation and consumption since their commercialization in 1994, and by 2019 had a cumulative cultivation area of 2.7 billion hectares.^2^ GM crops generated a commercial profit of US$18.9 billion in 2018.^3^ In addition to the economic value of GM crops, various benefits, such as the preservation of species diversity, protection of the environment, reduction of carbon dioxide emissions, and increase in farm income, have been reported.^2,3^ However, despite its usefulness, dialogs over the pros and cons of GM technology and products have not yet reached a consensus.^4^ Differences in opinion about GMO are reportedly based on differences in interests between producers, suppliers, and consumers as well as in scientific knowledge levels between experts and consumers.^5,6^ In particular, consumers’ lack of knowledge about GMO is accompanied by its fear, and ultimately causes consumers to lower their preference for GMO.^7^

It has been reported that diverse information regarding GMO, such as those relating to development, safety, risk evaluation, and safety management, will increase opportunities to provide consumers with more accurate and impartial information based on science, to enhance mutual understanding between developers and consumers.^8,9^ A study by,^10^ shows that knowledge could dampen the effects of fear and the consequent and almost automatically adverse behavior toward newly developed food purchases. Further, in a study by,^11^ targeting Greek consumers, it was found that accurate information on GM safety and GMO’s nutrition had a significant influence on their purchase intention.

The Act on the International Movement of Living Modified Organisms [LMO) enacted in 2014 in Korea, requires the relevant government organizations to provide information on GMO to enhance public awareness and understanding. Although several systems are in place to ensure safety and provide information on GMO, these policies do not guarantee the provision of accurate knowledge to consumers. In the study of,^12^ in order to improve the effectiveness of the mandatory labeling of foods containing GMO ingredients, the government should invest in education policy, targeting the elderly and individuals with lower levels of education. In a study by,^13^ GMO labeling was found to negatively influence consumers’ opinions and behavioral intentions if consumers did not have sufficient knowledge about the usefulness of GM foods. Nevertheless,^14^ found that consumers had a preference of enhanced labeling information related to GM foods and that such a preference significantly influenced their willingness to pay for GM foods. Moreover, a study by,^15^ found that consumers who were uncertain about the safety of GM foods tended to be more in favor of regulation.

Many studies have reported that the information provided poses problems for smooth dialogue as it is a one-sided communication that emphasizes safety, including the benefits that agricultural biotechnology provides, and the strictness of safety control and regulations.^16–18^ Communication on controversial scientific issues often raises concerns about the unknown, and a lack of trust can exacerbate fear, risk awareness, anxiety, and anger in people.^19^ The government’s provision of insufficient information on GMO causes the public to demand more information; meanwhile, reporters write speculative stories to meet such demands, resulting in exaggerated and distorted information.^16^ Indeed, media reports tend to focus on the risks, concerns, and uncertainties surrounding GMO.^20^ Such negative and exaggerated information in the mass media is magnified and reproduced through personal blogs and online communities, resulting in a vague anxiety in the public.^21^ A study by,^3^found that the combination of confusing science and social media produced a context that is ripe for misinformation to prevail and spread rapidly, even for issues such as GM food consumption that have reached the level of established science. The issue of trust and public acceptance of GM technology has been extensively debated, even as consumer safety concerns and distrust of food producers continue to grow due to poor communication about the safety of GM foods.^22^

Misinformation about major health issues, such as autism and vaccines, can affect public perception and policy.^23–25^ound that misinformation is widespread and reported that 88% of the websites surveyed often misrepresented information. Various studies have been conducted in different countries on consumer confidence and attitude toward GMO. It is generally agreed that consumers have limited knowledge of GMO. Thus, social trust (such as trust in agency or knowledge) may play a key role in their attitude toward GMO.^26–30^ Therefore, for consumers’ social trust and proper knowledge of GMO, the information channels through which they obtain such information is very important. Although GMO-related information does not always come directly from scientific sources, consumers tend to trust them over alternative sources.

### Conceptual Framework

**Figure 1.**
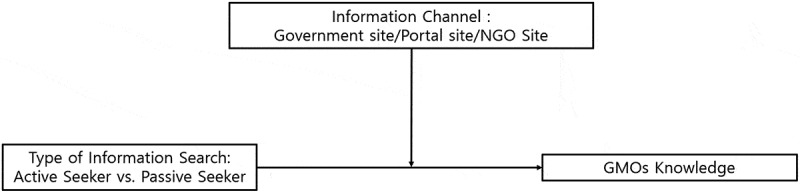
Conceptual Framework.

In this study, we explored how consumers’ type of information search and preferred channel for GMO information influenced their objective knowledge. To this end, the study divided search types for GMO information into active and passive seekers, and then examined how their knowledge differed, depending on the preferred information channel (i.e., government, portals, NGO sites) as shown in Figure 1. To achieve the purpose of this study, the following research questions were explored: (a) do the type of information search, information channel, and GMO knowledge differ according to demographics? (b) what are the main effects of the type of information search and information channel on GMO knowledge? and (c) what are the interactive effects of the type of information search and information channel on GMO knowledge?

### The Relationship between Type of Information Search and GMO Knowledge

Generally, in consumer science, active information seekers are defined as information-conscious, rational, influential, active, and elite groups.^31^ The consumption activity of active information seekers positively influences consumers who are comparatively passive and is the basis for the development of the overall market system.^32^

However, active information search does not always bring positive results to consumers. When more information is provided, consumers are more satisfied, but in reality, they can make worse purchase decisions with more information.^33^ According to,^34^ the complexity of information increases parallelly with increases in its amount, leading to information overload for consumers, which can have a negative impact on their product purchases.

Consumers in the GMO market, faced with information disseminated by various information providers, are inundated with information that is excessive, intentional, and ambiguous. Consumers in this information environment may feel confused in their search for information or selection of a product to purchase. Therefore, even if consumers go through many information retrieval processes, there is a possibility that the results are somewhat inefficient or unsatisfactory.,^35^observed that consumer confusion may be attributed to not only increases in the number of alternatives provided to the consumer, but also to increases in the information regarding the product,^36^ described information overload as the loss of control over a situation and the experience of being overwhelmed by information. Therefore, consumers who actively search for information about GMO may experience more confusion than consumers who do not, and it is expected that the objective GMO knowledge level of active information seekers may be lower than that of passive information seekers. Accordingly, hypothesis 1 was established based on the preceding studies.

***Hypothesis 1***. The objective level of knowledge of consumers about GMO will be different according to the type of information search.

### The Effect of Information Channel

In the market, consumers may face difficulties in not only the process of product selection but also in the process of information search, as they access information through various channels.^37^ When exposed to a large number of information channels or excessive information, consumers feel confused, such that they are unable to process the information smoothly and recognize their inability to utilize it effectively.^38^ According to a study by,^38^ mistrust confusion occurs mainly when it is difficult to determine which information is accurate among information obtained from different channels.

Information channels related to GMOs are also numerous and diverse, thus this information environment presents a new challenge for consumers. Among the various channels, it is highly likely that information is collected through channels that consumers trust relatively, and consumers’ knowledge and attitudes about GMOs may change depending on the information channel selected.

A study of U.S. consumers found that scientists, considered to be impartial evaluators, along with university research groups and medical professionals, were among the most reliable sources of GMO information. They were rated as more reliable than civic and environmental groups, farmers, the media, grocery stores, and industry groups.^39^ Studies of risk perception show that the public trusts experts (including university scientists, environmental groups, and consumer groups) more than the government, supermarkets, and corporate scientists, for sources of GMO information. When these expert sources agreed with government agencies on GMO, the government message seemed fairer and more authoritative, indicating thus that consumers trusted expert-backed messages more than information from non-experts.^40^

A study by,^41^targeting Italian consumers also found that consumers’ trust varied according to the source of information. The most credible source for information on biotechnologies was represented by consumers’ organizations (43%) that have continued to increment their consensus in recent years (26% in 2001 and 36% in 2006). Universities and institutes for scientific research were also recognized (13%) but much lesser than environmental organizations (25%); followed by public authorities (9%), religious organizations (5%), industries (4%), and lastly, political parties (1%).

According to a recent survey conducted in Korea on preference and trusted websites for collecting information, ‘NGO websites and blogs’ showed a 12.3% preference rate after ‘various web portals’ (53.4%). These had higher preference rates than ‘professional information websites’ (11.4%) and ‘academic websites’ (9.3%).^42^ However, for trusted websites, ‘various web portals’ (28.4%) had the highest rate, followed by ‘academic websites’ (19.1%), ‘professional information websites’ (18.2%), and ‘NGO websites and blogs’ (17.4%). In Korea, portal sites that provide various services, including search engines, are the most commonly used. Korean portal sites are more than just search engines, as they have become comprehensive media services, they are at the center of information exchange.^43^ The domination of the Internet by portals in Korea is a phenomenon not observed in other countries. Unlike portals, search engines such as Google, which only provide search results, have a significant share worldwide. However, search engines are shadowed by portals’ market-dominating power and do not exhibit such an influence in Korea.^44^ Understanding consumers’ current knowledge levels is essential to study their perceptions of GMO^45^ reported that most people believe they have some knowledge of GMO, but very few of them answered two questions related to the topic correctly, thereby demonstrating that people are generally overconfident about their knowledge of GMO.

In summary, the level of objective knowledge of consumers about GMO may vary depending on consumers’ preferred information channels. Moreover, if consumers were more actively seeking information through channels that provide untrustworthy information, the level of objective knowledge about GMO could be lowered. Accordingly, hypotheses 2 and 3 were established based on the preceding studies.

***Hypothesis 2.*** The objective level of knowledge of consumers about GMO will be different according to the information channel.

***Hypothesis 3.*** The relationship between the type of information search and GMO knowledge depends on the information channel.

## Methods

### Data

This study aimed to identify consumers’ needs and how they search for information on GMO, and find ways to provide information to them more efficiently in the future.

The survey was conducted on Korean men and women aged 19 or older using a web survey. The survey was conducted from March 26 to April 10, 2020 and the sampling was performed by gender, age, and region. Of the total 1,869 samples, 543 people with experience of voluntary online information collection were selected, and 1,326 samples with no experience of information collection for GMO were excluded. The main characteristics of the respondents are shown in Table 1.

**Table 1.** Characteristics of Participants

Characteristics	Frequency (%)
Gender	
Male	290 (53.4)
Female	253 (46.6)
Age	
20–29	108 (19.9)
30–39	143 (26.3)
40–49	126 (23.2)
50–59	117 (215)
60≤	49 (9.0)
Education	
High school	66 (12.8)
College	373 (72.1)
Graduate	78 (15.1)
Type of information search	
Active information seeker	274 (50.5)
Passive information seeker	269 (49.5)
Information Channel	
Government site	131 (25.4)
Portal site	237 (43.6)
NGO site	73 (14.1)
	m (S.D)
GMOs knowledge	4.63 (2.17)

### Measures

With regard to the type of information search, those who searched for information on GMO more than once a month were classified as active seekers; otherwise, they were classified as passive seekers.

Information channels presented in the questionnaire included government, portal, NGO, corporate, and personal sites. The three most preferred sites by respondents for searching GMO information were: government, portal, and NGO sites.

With regard to GMO knowledge, respondents answered 10 true-or-false questions related to objective facts, receiving one point for the right answer and zero for the wrong one. Therefore, GMO knowledge ranged from 0 to 10 points (supplementary data 1).

### Analysis

SPSS 21.0 (Chicago, IL) was used to conduct the analyses. A chi-squared test was employed to assess whether the respondent characteristics were related to the type of information search and information source. In addition, an analysis of variance (ANOVA) with the post-hoc Duncan test was conducted to examine the mean differences in GMO knowledge according to respondent characteristics. The general linear model (GLM) was used to examine the main effects of the type of information search and information source, in addition to examining their interaction effects on GMO knowledge. The GLM underlies most of the statistical analyses that are used in the applied and social sciences. It is the foundation for the t-test, analysis of variance (ANOVA), analysis of covariance (ANCOVA), and regression analysis.^46^ The GLM provides a general framework for a large set of models whose common goal is to explain or predict a quantitative dependent variable by a set of independent variables that can be categorical or quantitative. In this study, the GLM is an ANOVA procedure in which the calculations are performed using a least squares regression approach to describe the statistical relationship between two categorical predictors (i.e., type of information search and information channel) and a continuous response variable (i.e., GMO knowledge).^47^ Therefore, the purpose of GLM in this study is to verify the mean difference of the dependent variable according to the independent variable based on the F value. Further, the GLM is widely used in social sciences due to its suitability for verifying the interaction effect as well as the main effect in factorial design.^46^

## Results

### Type of Information Search according to Demographics

The results of the type of information search according to demographics are shown in Table 2; only age was statistically significant ($\chi^{2}=10.321,\;p=0.35)$. For active information seekers (AIS), the ratio of 40–49 years old was the highest; the ratio of 30–39 years old was the highest for passive information seekers (PIS). Overall, the age distribution of the entire sample and of AIS and PIS showed similar patterns.

**Table 2.** Type of Information Search According to Demographics

Characteristics	Type of information search		$\chi^{2}$	*p*
	AIS^1^ n (%)	PIS^2^ n (%)		
Gender				
Male	148 (51.0%)	142 (49.0%)	.556	.456
Female	121 (47.8%)	132 (52.2%)		
Age				
20–29	56 (20.4%)	52 (19.3%)	10.321	.035
30–39	63 (23.0%)	80 (29.7%)		
40–49	78 (28.5%)	48 (17.8%)		
50–59	53 (19.3%)	64 (23.8%)		
60≤	24 (8.8%)	25 (9.3%)		
Education				
High school	32 (12.9%)	34 (12.6%)	.016	.992
College	179 (72.2%)	194 (72.1%)		
Graduate	37 (14.9%)	41 (15.2%)		

Note: ^1^Active information seeker, ^2^Passive information seeker

### Information Channels according to Demographics

The results of the information channel according to demographics are shown in Table 3; no statistically significant differences in gender, age, and education were found.

**Table 3.** Information Channel According to Demographics

Characteristics	Information Channel			$\chi^{2}$	*p*
	Government site N (%)	Portal site N (%)	NGO site N (%)		
Gender					
Male	60 (27.1%)	118 (53.4%)	43 (19.5%)	3.241	.391
Female	71 (32.3%)	119 (54.1%)	30 (13.6%)		
Age					
20–29	30 (36.6%)	38 (46.3%)	14 (17.1%)	6.398	.603
30–39	34 (28.6%)	62 (52.1%)	23 (19.3%)		
40–49	31 (32.0%)	50 (51.5%)	16 (16.5%)		
50–59	24 (24.2%)	62 (62.6%)	13 (13.1%)		
60≤	12 (24.2%)	25 (56.8%)	7 (15.9%)		
Education					
High school	16 (28.1%)	33 (57.9%)	8 (14.0%)	2.287	.683
College	96 (29.8%)	168 (52.2%)	58 (18.0%)		
Graduate	19 (30.6%)	36 (58.1%)	7 (11.3%)		

### GMO Knowledge according to Demographics

The results of GMO knowledge according to demographics are shown in Table 4; only age was statistically significant (*F* = 2.957, *p* = .20). The 30–39 group had the highest score, and the 60 ≤ group had the lowest score.

**Table 4.** GMO Knowledge According to Demographics

Characteristics	GMOs knowledge m (S.D)	t/F	p
Gender			
Male	4.64 (2.28)	.112	.911
Female	4.62 (2.03)		
Age			
20–29	4.56 (2.20)^ab^	2.957	.020
30–39	5.05 (2.02)^b^		
40–49	4.25 (2.34)^a^		
50–59	4.78 (2.06)^ab^		
60≤	4.20 (2.16)^a^		
Education			
High school	4.65 (1.82)	.497	.608
College	4.91 (1.91)		
Graduate	4.83 (1.95)		

Note: ^A,ab,b^In the Duncan test, the significance of the mean difference of each group was verified at the 0.05 level, and the degree of the mean of each group was expressed as a ≤ ab ≤ b

### Effects of Type of Information Search and Information Channel on GMO Knowledge

The results of main effects and interactive effects of the type of information search and information channel on GMO knowledge are presented in Table 5. The main effects of all variables were statistically significant. Therefore, hypotheses 1 and 2 were supported.The interactive effects between the type of information search and all information channels were statistically significant, therefore, hypothesis 3 was supported.

**Table 5.** Effects of Type of Information Search and Information Channel on GMOs Knowledge

	Estimated mean (S.E.)	SS	df	F	*P*	*η_p_^2^*
Type of information search		17.428	1	4.156	.042	.008
Active information seeker	5.058 (.271)					
Passive information seeker	5.844 (.274)					
Government site		89.130	1	21.254	.000	.038
Yes	6.076 (.034)					
No	4.825 (.137)					
Portal site		64.583	1	15.401	.000	.028
Yes	5.927 (.285)					
No	4.974 (.150)					
NGO site		57.539	1	13.721	.000	.025
Yes	6.032 (.333)					
No	4.869 (.112)					
Type of information search × Government site		65.489	1	15.617	.000	.028
Type of information search × Portal site		85.775	1	20.454	.000	.037
Type of information search × NGO site		65.897	1	15.714	.000	.029

**Figure 2.**
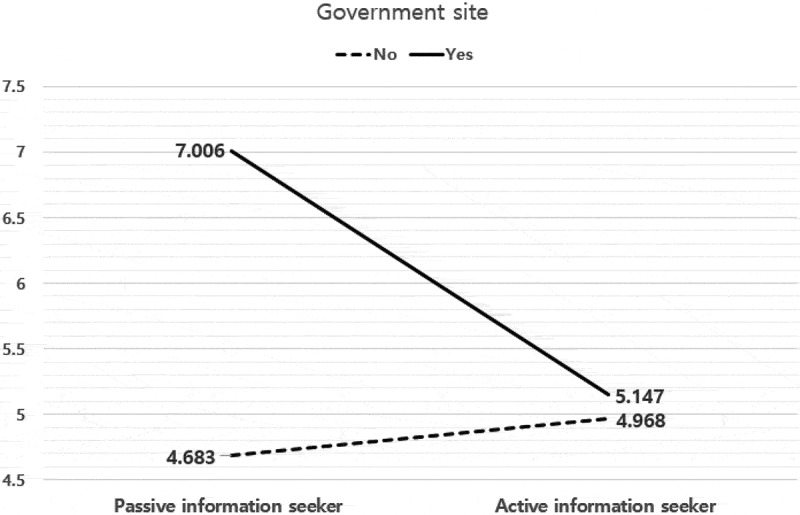
Effects of the Type of Information Search and Government Site on Consumers’ GMOs Knowledge.

The interactive effect between the type of information search and the government site was statistically significant (*F* = 15.617, *p* ≤05, *η_p_^2^ *= .028); the results are shown in Figure 2. The GMO knowledge score of the group that preferred government sites was found to be higher in both PIS and AIS, than those that preferred other sources. Further, in the case of the group that preferred the government site, the GMO knowledge score of PIS was higher than that of AIS; however, in the group that did not prefer the government site, the GMO knowledge scores of PIS were lower than that of AIS.)

**Figure 3.**
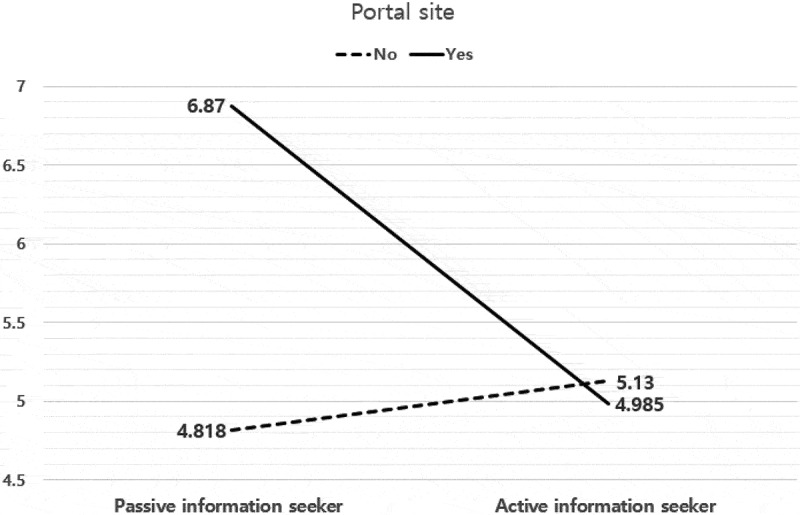
Effects of the Type of Information Search and Portal Site on Consumers’ GMOs Knowledge.

The interactive effect between the type of information search and the portal site was statistically significant (*F* = 20.454, *p* ≤000, *η_p_^2^ *= .037); the results are shown in Figure 3. In the PIS group, the GMO knowledge score of the group that preferred portal sites was higher than the score of the group that preferred other sites. However, in AIS, the opposite was true, that is, the GMO knowledge score of the group that preferred portal sites was lower than the score of the group that preferred other sites.

**Figure 4.**
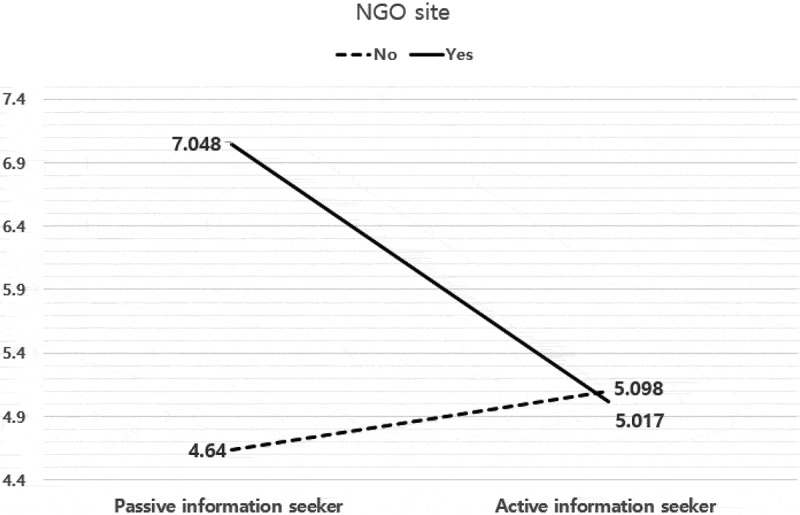
Effects of the Type of Information Search and NGO site on Consumers’ GMOs Knowledge.

The interactive effect between the type of information search and the NGO site was statistically significant (*F* = 15.714, *p* ≤ .000, *η_p_^2^* = .014); the results are shown in Figure 4. In the PIS group, the score of GMO knowledge of the group that preferred NGO sites was higher than the score of the group that preferred other sites. However, in AIS, the opposite was true.

## Discussion

GMOs comprise products that are difficult to assess in terms of profits and risks; therefore, information about these is collected through a variety of information channels,^48^ revealed that the information channel can play a significant role in advancing or discouraging consumer acceptance of GMOs and foods. Therefore, in this study, to investigate the moderating effect of the information channel in the relationship between the type of information search and GMO knowledge, we examined the main effects and interactive effects of the type of information search and information channel on GMO knowledge.

First, the main effect of the type of information search was statistically significant. As the GMO knowledge score of AIS is lower than that of PIS, and these results are consistent even when considering the moderating effect of the information channel. Therefore, consumers are currently experiencing confusion due to too much information through various information channels. In traditional consumer information research, consumers who actively search for information make more rational decisions.^31^ However, although a lot of information is delivered to consumers, the accuracy and objectivity of this information are not guaranteed; thus, the more consumers search for information, the lower their objective knowledge about GMO. These results are consistent with the findings of previous research. According to,^49^ too much information surrounding a product or service disturbs the consumer by forcing them to engage in more complex thinking. This, in addition to the fact that it is difficult to compare and value the information when it is superfluous, leaves the consumer unsatisfied, insecure regarding what choice to make, and more prone to delay decision-making. In a study by,^50^ consumers who actively search for information when they select technologically complex products, such as notebook computers, were found to experience more confusion than consumers who did not actively seek information,^51^ reported that consumer confusion also occurs when consumers lack searching skills, but generally occurs when there is excessive information.

Second, regarding the main effects of information channels, it was found that when government, portal, and NGO sites were preferred, the level of GMO knowledge was higher than that of consumers who did not prefer these. However, these results are somewhat different when considering the interaction effect with the type of information search.

Regarding the interactive effect between the type of information search and the government site, the group that preferred the government site in both the PIS and the AIS group had a higher GMO knowledge score than the group that preferred other sites; this is attributed to the objectivity and accuracy of the information provided by the government site, which tends to provide consumers with information that is relatively more verifiable and unbiased. According to a study by,^52^ and^53^ the information on government sites has higher levels of trust among consumers compared to other sites (e.g., portals, companies, NGOs) in Korea. Previous research shows that there may be a need for third-party verifiable information on GMOs (i.e., government sites), so that consumers do not have to rely on the information from biotechnology companies and environmental groups.^54^ Research on organic foods reached a similar conclusion, in that there may be benefits of having an independent, third-party monitor to help reduce false claims made by interested parties.^55^ Additionally,^56^ showed that expert organizations highlighting scientific consensus on GM food safety reduced consensus misperceptions among the public, leading to lower GMO misperceptions and boosting related consumption behaviors. Given that GMOs are directly related to the health of consumers, and that bioengineering is a complex process, most people do not know the intricate details of this process. The government site that provides introspective and verified information, thus, enhances the knowledge of consumers rather than providing biased information, emphasizing both the benefits and risks of GMOs.

Although the information on the government website is relatively objective and accurate, the GMO knowledge score of AIS is lower than the PIS score in the group that prefers the government website. In the results regarding portal and NGO sites, the score of AIS in the group that preferred these sites was lower than that of PIS, and even lower than that of AIS in the group that did not prefer these sites. Since consumers in the recent information environment are exposed to information through various channels, they may experience difficulties not only in the process of product selection but also in the process of information search.^51^ According to the study of,^57^ it was found that the degree of consumer confusion differs according to the extent to which a consumer searches for information and the type of information channel. Their empirical results revealed that even when third-party information was used, the confusion of consumers increased.^57^

Considering the results of portal and NGO sites, in the AIS group, the GMO knowledge scores of the groups that preferred these sites were lower than those of the groups that did not. It is worth paying attention to the characteristics of the portal and NGO sites, and the inaccuracy of the information provided by these sites.

The diversification of information channels has the advantage of providing a wealth of information to consumers, but confusion arises due to difficulties in information search or overload.^58,59^ mphasizes the concerns about whether information through information channels can be trusted. Excessive information is being provided chaotically through multiple channels, making it difficult to find the necessary information, and problems such as difficulty in clearly recognizing the properties of the channel occur.^51^

In Korea, portal sites that provide a variety of services, including functioning as search engines, are most often used. In Korea, all information is exchanged on portal sites.^43^ Specifically, Korean portal sites employ an integrated search model, which refers to a search method that categorizes and displays all related data, such as cafe/blogs, knowledge searches, dictionary, images, videos, music, latest news, regions, books, shopping, and search terms.^60^ Therefore, when consumers search for information on portals, they tend to scan all the information provided by various sources (e.g., cafe/blog, knowledge search, latest news, shopping), regardless of their credibility in the medium.^44^ For this reason, the accuracy of GMO information provided by portal sites is inevitably disregarded. Furthermore, NGOs, such as Greenpeace, oppose agricultural biotechnology and maintain that biotech foods cause allergic reactions, harm the environment, and increase the power of multinational companies. Additionally, consumer advocates and a wide range of environmental and food safety groups have mounted active campaigns against biotech foods.^61,62^ ound that when consumers were presented with both positive and negative information on food irradiation, the negative information dominated consumers’ decision-making. This was the case despite the fact that the negative information source was identified as a consumer advocacy group, and the information was written non-scientifically. Therefore, it is possible that the information on the NGO site reflects the NGOs views without scientific corroboration. Ultimately, the portal and NGO sites tend to provide unverified or biased information about GMOs; such information is confusing for groups actively seeking information. This could explain why the level of knowledge of AIS is lower than that of PIS in this study. Therefore, the importance of the channel of biotechnology information should be emphasized in relation to accurate information and consumers’ accurate understanding.^63^ Finally, with different channels offering information to consumers, the source of consumer education, not just the education itself, has emerged as a crucial factor in the accurate understanding of biotech foods.^63^ As,^54^ argued, independent, third-party information improves consumers’ welfare in the biotechnology environment.

This study is significant in the following respects. First, by verifying the empirical result that consumers who actively seek information have lower levels of GMO knowledge than consumers who passively seek information, it can be confirmed that Korean consumers are confused about a lot of information related to GMOs. Second, compared to government sites that provide relatively objective and accurate information, the importance of information channels is empirically confirmed by revealing that consumers who prefer portal and NGO sites may lower their knowledge of GMOs if they actively collect information.

This study clarified the importance of the information channel, but there are some limitations, as it was a secondary analysis. First, most of the participants in the study had a high level of education, with a high school graduation or higher. Therefore, in future research, it is necessary to examine the relationship between academic background and biotechnology knowledge by broadening the demographic pool of participants. Second, in this study, information channels were limited to government, portal, and NGO sites. Therefore, it is necessary to include more diverse channels in future studies.

## Supplementary Material

Supplemental Material
