# Supplementary material for: The moderating effect of information channel on the relationship between type of information search and knowledge of genetically modified organisms
Source: GM Crops Food. 2022 Jan 30;13(1):26–37. doi: 10.1080/21645698.2021.2015272 (PMC8890432; doi:10.1080/21645698.2021.2015272)
Supplement: Supplemental Material [file KGMC_A_2015272_SM8797.docx]

**Supplementary data 1**

Questions for GMO knowledge

| Items | True | False | Don’t know |
| --- | --- | --- | --- |
| 1. GM crops are injected with one to four foreign genes, resulting in very small genetic changes compared to conventional breeds | ● |  |  |
| 2. GM beans contain genes, but non-GM beans do not. |  | ● |  |
| 3. GM crops and foods can alter the genes of the person who consumes them. |  | ● |  |
| 4. GM crops with fish genes, taste like fish. |  | ● |  |
| 5. It is impossible to transplant animal genes into plants. |  | ● |  |
| 6. About 80% of GM crops imported into Korea are for animal feed. | ● |  |  |
| 7. GM agricultural products must pass safety screening to be grown or consumed. | ● |  |  |
| 8. More than 80% of the world's beans in cultivation are GM beans. | ● |  |  |
| 9. Currently, GM agricultural products are grown commercially in Korea. |  | ● |  |
| 10. In Korea, GM foods are labelled. | ● |  |  |

※ ● indicates collect answer.
